# Assessment of Transboundary PM2.5 from Biomass Burning in Northern Thailand Using the WRF-Chem Model

**DOI:** 10.3390/toxics12070462

**Published:** 2024-06-26

**Authors:** Kevalin Inlaung, Chakrit Chotamonsak, Ronald Macatangay, Vanisa Surapipith

**Affiliations:** 1 Office of Research Administration, Chiang Mai University, Chiang Mai 50200, Thailand; kevalin.in@cmu.ac.th; 2 Environmental Science Research Center, Faculty of Science, Chiang Mai University, Chiang Mai 50200, Thailand; 3 National Astronomical Research Institute of Thailand (Public Organization), Chiang Mai 50180, Thailand; ronmcdo@gmail.com; 4 Asian Disaster Preparedness Center, Bangkok 10400, Thailand; vanisas@gmail.com

**Keywords:** transboundary air pollution, PM2.5, WRF-Chem model, biomass burning

## Abstract

Air pollution, particularly PM2.5, poses a significant environmental and public health concern, particularly in northern Thailand, where elevated PM2.5 levels are prevalent during the dry season (January–May). This study examines the influx and patterns of transboundary biomass burning PM2.5 (TB PM2.5) in this region during the 2019 dry season using the WRF-Chem model. The model’s reliability was confirmed through substantial correlations between model outputs and observations from the Pollution Control Department (PCD) of Thailand at 10 monitoring stations. The findings indicate that TB PM2.5 significantly influences local PM2.5 levels, often surpassing contributions from local sources. The influx of TB PM2.5 began in January from southern directions, intensifying and shifting northward, peaking in March with the highest TB PM2.5 proportions. Elevated levels persisted through April and declined in May. Border provinces consistently exhibited higher TB PM2.5 concentrations, with Chiang Rai province showing the highest average proportion, reaching up to 45%. On days when PM2.5 levels were classified as ‘Unhealthy for Sensitive Groups’ or ‘Unhealthy’, TB PM2.5 contributed at least 50% to the total PM2.5 at all stations. Notably, stations in Chiang Rai and Nan showed detectable TB PM2.5 even at ‘Very Unhealthy’ levels, underscoring the significant impact of TB PM2.5 in the northern border areas. Effective mitigation of PM2.5-related health risks requires addressing PM2.5 sources both within and beyond Thailand’s borders.

## 1. Introduction

Air pollution, particularly PM2.5 (fine particulate matter), is a significant environmental threat with local to regional impacts due to the transboundary movement of pollutants. PM2.5 has a long atmospheric residence time, allowing its dispersal over extensive distances, influenced by factors such as topography, air mass dynamics, and particulate matter properties. The consequences include compromised human health, reduced visibility, disruptions to daily life, and ecological damage, highlighting underlying vulnerabilities [1].

Southeast Asia (SEA) frequently experiences haze episodes caused primarily by the open burning of crop residues, slash-and-burn agriculture, and forest and peatland fires. Indonesia is a major source, but other countries, including Malaysia, Singapore, Myanmar, Laos, Vietnam, Cambodia, and Thailand, also contribute to transboundary haze. The 2019 SEA smoke haze event was particularly severe, exacerbated by El-Nino Southern Oscillation (ENSO) conditions [2].

Efforts to prevent and mitigate transboundary air pollution from biomass burning include regional and local agreements, measures, and regulations, utilizing science, technology, and research [3]. Despite these efforts, research consistently shows significant associations between haze exposure and acute adverse health outcomes, including respiratory, cardiovascular, and neurological issues [4]. In Thailand, the population faces this crisis almost annually, with increased chronic respiratory diseases over the last two decades [1,5,6,7]. Most PM2.5 solutions are short-term interventions, addressing the problem only during or after its occurrence, with insufficient focus on the earlier phases of threat response [8]. Complex pollution source governance and inadequate academic research limit preparedness and mitigation effectiveness, increasing social vulnerability and community instability.

This study aims to investigate the impact of transboundary biomass burning PM2.5 pollution in northern Thailand, focusing on its sources, distribution, and potential implications. The research includes a spatial pattern analysis of transboundary biomass burning PM2.5 across nine provinces in northern Thailand, characterizes the properties of this pollution, and classifies its impacts on local air quality and public health.

## 2. Methodology

### 2.1. Study Area

**Figure 1:**
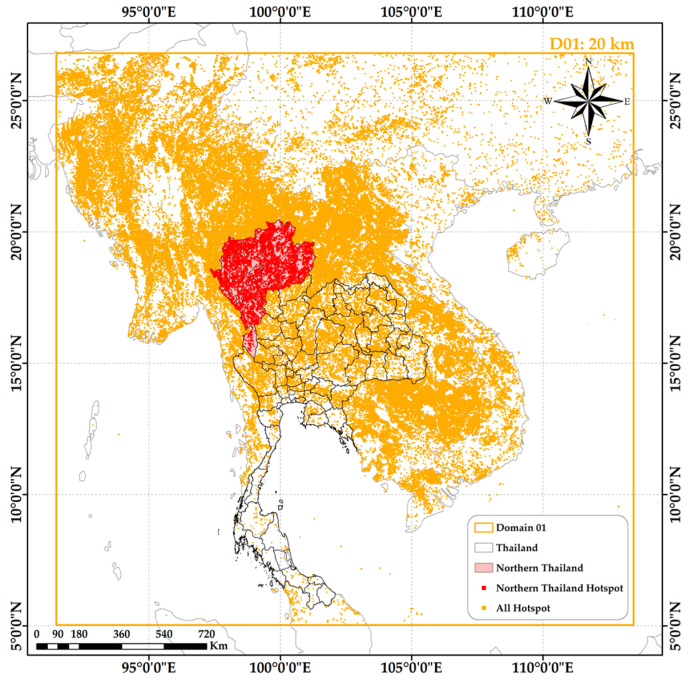
Study area (northern Thailand) and the surrounding area within WRF-Chem Domain 01, including the MODIS fire hotspot data from January to May 2019.

This study focuses on northern Thailand, a region with a high incidence of haze. This study covers nine provinces, Chiang Rai, Chiang Mai, Nan, Phayao, Phrae, Mae Hong Son, Lampang, Lamphun, and Tak, as shown in Figure 1. These provinces are characterized by complex mountainous formations that significantly influence their topography. Northern Thailand predominantly experiences a Tropical Savanna Climate (Aw) and a Tropical Monsoon Climate (Am) according to the Köppen climate classification system. The region’s climate is marked by three distinct seasons: hot, cool–dry, and rainy. The total area of the northern region is approximately 105,503.82 km^2^. The surrounding area, denoted WRF-Chem Domain 1 (D01) in Figure 1, extends across Southeast Asia, including Thailand, Myanmar, Laos, Vietnam, and Cambodia, and portions of India, China, Malaysia, and Indonesia, and is characterized by a tropical climate.

Local activities and topographical and meteorological conditions, such as temperature inversions, exacerbate PM2.5 concentrations in northern Thailand. Biomass burning both within and outside the area significantly contributes to the haze crisis [9]. This study emphasizes the complex dynamics of transboundary biomass burning PM2.5, arising from multiple factors, including variable meteorological conditions, diverse sources of biomass burning, and the interactions between local and transboundary pollutants [1,10,11].

### 2.2. Study Period

The study period is defined as 1 January to 31 May 2019, coinciding with the dry season, during which severe haze incidents are most frequent in northern Thailand. During this time, daily PM2.5 concentrations ranged from moderate to hazardous levels. Haze episodes in this region follow a distinct seasonal pattern, with air pollution trends from 2004 to 2018 showing significant increases, peaking in March [12]. Meteorological conditions such as low wind speed, high humidity, and elevated temperatures contribute to high PM2.5 levels [13]. Future projections for 2020–2029 indicate that PM2.5 concentrations will increase significantly during the dry season, with values ranging from 40 to 400 µg/m^3^ [14]. The primary source of PM2.5 is biomass burning, particularly open burning in forests and agricultural areas, which surpasses secondary formation from photochemical processes or other anthropogenic emissions [7,15,16,17].

### 2.3. Data Utilized

#### 2.3.1. Fire Hotspots

A dataset of fire hotspots, specifically those generated by biomass burning activities, was obtained from the active fire data provided by the Moderate Resolution Imaging Spectroradiometer (MODIS) [18]. This sensor instrument is aboard the AQUA/TERRA satellites. The dataset, provided by the National Aeronautics and Space Administration (NASA), is available in shapefile format. The selected dataset covers the area designated D01 in Figure 1.

#### 2.3.2. PM2.5 Observations

**Figure 2:**
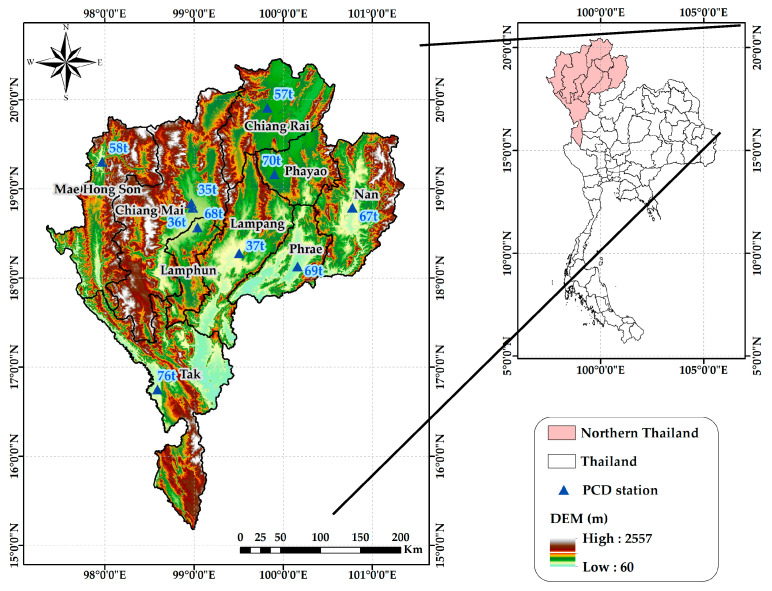
Location of the PCD air quality monitoring stations in the study area (northern Thailand).

This study utilizes an observation dataset consisting of hourly PM2.5 concentration measurements obtained from air quality monitoring stations operated by the Pollution Control Department (PCD) of Thailand. The PCD network comprises 10 monitoring stations strategically distributed across the 9 northern Thailand provinces, as detailed in Table 1 and Figure 2.

### 2.4. WRF-Chem Model Utilization

#### 2.4.1. Model Setup

We utilized the WRF-Chem model version 3.4.1 to simulate TB PM2.5. WRF-Chem (Weather Research and Forecasting model coupled with Chemistry) is a well-established tool for air quality and atmospheric chemistry studies [19] The parameter settings for the simulations were chosen based on previous studies and sensitivity analyses to ensure the model accurately captured the local and regional atmospheric processes and are detailed in Table 2 [20].

#### 2.4.2. Emission Inventories

We incorporated both local and regional emission inventories into the model. The EDGAR-HTAP dataset was used to analyze the transboundary movement of air pollutants, providing a comprehensive mosaic of regional and global emission grids [27]. Meanwhile, biogenic sources were calculated online using the Model of Emissions of Gases and Aerosols from Nature (MEGAN) [28]. The biomass burning emissions were calculated using daily FIRMS MODIS fire hotspots with the 3BEM algorithm [29] in the PREP-CHEM-SRC preprocessing program [30]. These inventories provide detailed information on emission rates and species profiles necessary for accurate simulations.

#### 2.4.3. Simulation Scenarios

We conducted WRF-Chem simulations for two scenarios.

Case 1 Scenario (All Emissions): This scenario included both local and transboundary emissions to represent the total PM2.5 concentrations. Case 2 Scenario (Local Biomass Burning Emissions Only): This scenario included all emissions except for the transboundary biomass burning emissions, representing only the local contributions to biomass burning PM2.5. The difference in PM2.5 concentrations between these scenarios represents the contribution of biomass burning TB PM2.5. While we acknowledge that monitoring stations cannot distinguish between localized PM2.5 and TB PM2.5, our simulation design ensures that the observed differences are primarily due to variations in hotspot inputs. This is achieved by keeping all other variables constant, thus isolating the impact of transboundary biomass burning emissions.

#### 2.4.4. Validation

To ensure the accuracy and reliability of the WRF-Chem model in simulating PM2.5 concentrations, a comprehensive validation was performed using ground-based PM2.5 observations from monitoring stations across nine provinces. This validation process is crucial for assessing the model’s performance in representing real-world air quality conditions and for identifying any systematic biases or errors. By comparing the model outputs with actual observations, we can better understand the strengths and limitations of the model, ultimately leading to improved air quality forecasts and management strategies.

The rationale for selecting the statistical metrics is as follows. The correlation coefficient (r) was employed to assess the strength and direction of the linear relationship between the observed and predicted PM2.5 concentrations. A higher correlation coefficient indicates a stronger agreement between model predictions and actual observations (range: −1 to 1), thus validating the model’s reliability. The Root Mean Square Error (RMSE) was selected to quantify the average magnitude of errors between the predicted and observed values. The RMSE is particularly sensitive to larger errors (best when RMSE = 0), which is crucial in air quality studies where episodes of high pollution have significant health implications. The mean bias was used to evaluate the overall bias in model predictions (best when bias = 0). It indicates whether the model systematically overestimates or underestimates observed values, thereby providing insights into systematic deviations in model performance.

The calculation of these metrics is as follows. The *RMSE* was calculated using the following formula:

(1) $$RMSE=\sqrt{\frac{1}{N}\sum_{i=1}^{N}{\left(P_{i}-O_{i}\right)}^{2}},$$

where *N* is the number of observations, *P_i_* is the model predicted value, and *O_i_* is the observed value.

The mean bias was calculated using the following formula:

(2) $$Mean\;Bias=\frac{1}{N}\sum_{i=1}^{N}\left(P_{i}-O_{i}\right)$$

where *N* is the number of observations, *P_i_* is the predicted value, and *O_i_* is the observed value.

These metrics were computed for each monitoring station to provide the station-based assessment of the model’s performance. This thorough validation process ensures that the model accurately represents the observed PM2.5 levels, accounting for both transboundary and local sources. Utilizing these statistical metrics enables a robust and comprehensive evaluation of the WRF-Chem model’s capability to simulate PM2.5 concentrations, which is critical for guiding air quality management and policymaking decisions.

#### 2.4.5. Calculation of Biomass Burning TB PM2.5

The biomass burning TB PM2.5 was calculated by subtracting the PM2.5 concentrations in the Case 2 scenario, which includes all emissions, except for biomass burning within the northern area, from the concentrations in the Case 1 scenario, which includes all emissions. This approach isolates the impact of biomass burning transboundary sources on local PM2.5 concentrations.

#### 2.4.6. Analysis

The spatial and temporal distribution of TB PM2.5 was analyzed to identify hotspots and periods of high TB PM2.5. This analysis helps with understanding the dynamics of transboundary air pollution and its impact on northern Thailand.

### 2.5. Quantification of Temporal Persistence and Health Risks of Transboundary PM2.5

To assess the health risks associated with biomass burning TB PM2.5 concentrations, we utilized the criteria established by the United States Environmental Protection Agency (US EPA). This framework categorizes PM2.5 concentrations into three distinct levels based on their potential impact on sensitive population groups: 35.4 µg/m^3^ (Unhealthy for Sensitive Groups), 55.4 µg/m^3^ (Unhealthy), and 150.4 µg/m^3^ (Very Unhealthy). Our methodology involved quantifying the number of days on which the biomass burning TB PM2.5 concentrations exceeded these established thresholds. Additionally, we incorporated percentiles (25%, 50%, 75%, and 90%) to assess the prevalence of these exceedances across the ten monitoring stations. This comprehensive approach allowed for a detailed evaluation of both the frequency and severity of TB PM2.5 pollution events, providing critical insights into potential health risks for the affected populations.

## 3. Results

### 3.1. WRF-Chem Model Evaluation

**Figure 3:**
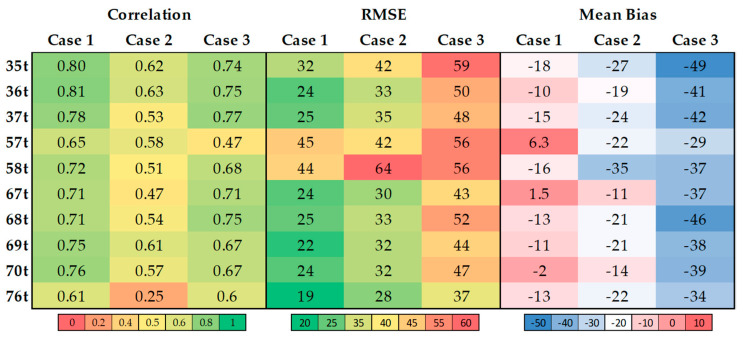
Statistical relationship heatmap between observed and model data for 10 PCD stations. The coefficient r represents, in the range of 0–1, a color gradient from red to green (r = 1 refers to perfect association); the RMSE represents, in the range of minimum–maximum value, the color gradient from green to red; and the mean bias represents, in the range of minimum–maximum value, the color gradient from blue to red (optimal RMSE and bias when being 0).

The relationship analysis in Figure 3 shows moderate to high positive correlation coefficients (r) [31] between observed data and model outputs in Case 1 (PM2.5 from all biomass burning hotspots). This suggests that the model effectively captures the trends in the observed data, indicating their reliability as representative indicators.

A similar pattern is observed for Case 3 (TB PM2.5), where a consistent positive correlation is evident between the observed data and model outputs. Most monitoring stations exhibit significant r values in Case 3, comparable to those in Case 1, indicating a strong positive relationship. This trend highlights the alignment between the modeled TB PM2.5 and the observed data, suggesting that TB PM2.5 significantly influences localized PM2.5 concentrations within the study area. Although station 57t shows a less pronounced relationship, it still indicates that this Chiang Rai location is affected by TB PM2.5 at similar levels to local sources.

Notably, r values in Case 3 consistently exceed those in Case 2 (PM2.5 from northern region biomass burning hotspots) across most monitoring stations, indicating that TB PM2.5 has a greater influence on observed PM2.5 concentrations than local biomass burning sources.

The Root Mean Square Error (RMSE) analysis, which measures the discrepancies between the model outputs and observed data, shows variance across the datasets. The mean bias analysis reveals systematic model over- or underestimation when compared to the observed data. In Case 1, the data variance is lowest among the monitoring stations, indicating minimal error. However, most stations show model underestimation, except for stations 57t (Chiang Rai) and 67t (Nan), where the model overestimates PM2.5 concentrations.

Comparing Case 2 and Case 3, Case 3 demonstrates greater variability across monitoring stations and shows model underestimation. In contrast, Case 2 exhibits lower variability and consistent model underestimation across all stations. This suggests that the observed data include contributions from diverse sources beyond biomass burning, such as additional PM2.5 emissions, resulting in higher observed PM2.5 concentrations than the model predictions.

### 3.2. Spatial Pattern Analysis of Transboundary Biomass Burning PM2.5 in Nine Northern Provinces of Thailand

**Figure 4:**
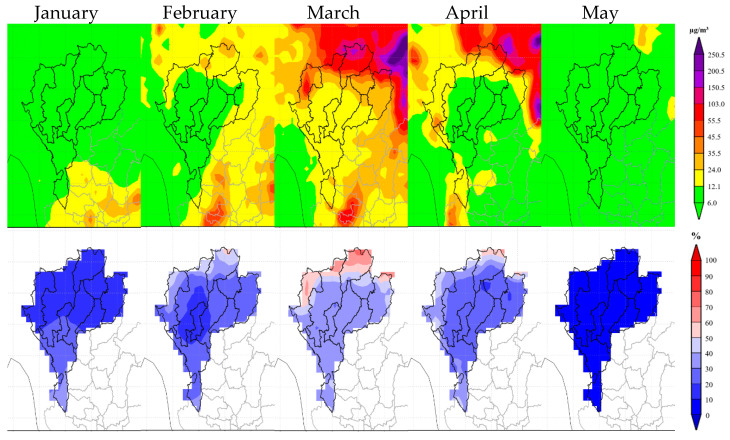
Monthly averaged TB PM2.5 concentrations in units µg/m^3^ (**upper**) and percentages (**lower**) from January to May 2019.

As depicted in Figure 4, this study highlights the persistent influx of transboundary biomass burning PM2.5 (TB PM2.5) into the designated area over the entire five-month period. PM2.5 concentrations reached notably Unhealthy levels, particularly from March through April. This timeframe witnessed a surge of over 50% in TB PM2.5 entering the study area. Provinces situated along the border experienced the most severe effects of TB PM2.5, marked by prolonged exposure and elevated PM2.5 concentrations. These provinces, located near international borders, are primary targets for TB PM2.5 influence, underscoring the vulnerability of borderlands to increased PM2.5 levels.

Analysis of monthly data reveals that during January, TB PM2.5 concentrations predominantly remain within a good level (green: 0–12 µg/m^3^). TB PM2.5 percentages stay below 40%, with the majority ranging between 10% and 20%. However, TB PM2.5 moderate levels (yellow: 12.1–35.4 µg/m^3^) are observed in the southern part of the study area. These levels escalate to Unhealthy for Sensitive Groups levels (orange: 35.5–55.4 µg/m^3^) in certain surrounding locations. The spatial pattern of this impact suggests a trajectory originating from the southern and southwestern directions. 

In February, there was an observed increase in TB PM2.5 concentrations compared to the previous month. While most of the study area maintains TB PM2.5 in good levels, the northern region exhibits elevated concentrations. Certain areas within Mae Hong Son, Chiang Mai, Chiang Rai, Phayao, Nan, and Phrae provinces experience moderate levels to Unhealthy for Sensitive Groups levels. The TB PM2.5 comprises 20% to 30% of the study area’s PM2.5 concentrations, remaining below the 50% threshold. External areas also demonstrate an increase, particularly in the southeast, where TB PM2.5 approaches Unhealthy levels. This surge is likely due to the influx of TB PM2.5 from northern, eastern, and southern sources.

March is considered a pivotal point of the study area, marked by the highest recorded TB PM2.5 concentrations. The majority of the region exhibits TB PM2.5 in moderate levels. However, concentrations escalate towards the north, reaching Unhealthy for Sensitive Groups levels and transitioning to Very Unhealthy levels (purple: 150.5–250.4 µg/m^3^). Mae Hong Son, Chiang Mai, and Phayao provinces demonstrate particularly Unhealthy levels (red: 55.5–150.4 µg/m^3^), while Nan and Chiang Rai provinces experience Very Unhealthy levels. Notably, Chiang Rai province records reaching 70% TB PM2.5 occurrence. The areas with high TB PM2.5 concentrations were found exceeding 50% TB PM2.5, while the areas of moderate concentration exhibited a 30–50% range of TB PM2.5. This concentration peak primarily affects the northern and western border sections of the study area.

In April, the study area continued to exhibit relatively high concentrations of TB PM2.5, with a spatial distribution similar to the previous month. While most of the study area experienced TB PM2.5 concentrations within a good level, elevated concentrations were observed in international border provinces. Specifically, Tak, Mae Hong Son, Chiang Mai, and Phayao provinces demonstrated concentrations ranging from moderate levels to Unhealthy for Sensitive Groups levels. Chiang Rai and Nan provinces exhibited areas reaching Unhealthy levels. Of note, areas in the northeast outside the study area showed significantly higher TB PM2.5 concentrations, extending to Very Unhealthy levels. Percentagewise, most areas registered TB PM2.5 proportions between 30% and 50%, with localized areas in Chiang Rai and Nan provinces exceeding 50–60%. In May, TB PM2.5 influence decreased, with concentrations falling within the good levels throughout the study area and percentages declining below 10%. However, the northern border region remained an exception, maintaining TB PM2.5 concentrations reaching Unhealthy for Sensitive Groups levels.

### 3.3. Characteristics of Transboundary Biomass Burning PM2.5 in Nine Northern Provinces of Thailand

The model simulations of transboundary biomass burning PM2.5 (TB PM2.5) were averaged for each province within the study area (Table 3). Throughout the study period, varying levels of TB PM2.5 affected each province from surrounding areas. Chiang Rai province exhibited the highest amount of TB PM2.5, with an average concentration of 29.41 µg/m^3^. Notably, TB PM2.5 comprised an average of 45% of all PM2.5 in this province. While other provinces displayed lower average TB PM2.5 concentrations, Mae Hong Son province had a secondary high proportion of TB PM2.5, averaging 37%. Lamphun province recorded the lowest average TB PM2.5 levels (7.67 µg/m^3^) and the lowest proportion, with TB PM2.5 constituting an average of 22% of all PM2.5.

Among the individual months, March displayed the highest TB PM2.5 concentrations throughout the study period. Chiang Rai province consistently exhibited the most significant TB PM2.5 concentrations, averaging 86.53 µg/m^3^. This value constitutes 68% of the overall TB PM2.5 average, indicating that a substantial portion of Chiang Rai’s PM2.5 originates from transboundary sources. Notably, Chiang Mai, Mae Hong Son, Phayao, and Nan provinces also displayed TB PM2.5 proportions exceeding 40%. Conversely, Lamphun province exhibited the lowest average TB PM2.5 concentration (15.36 µg/m^3^), representing only 35% of its total PM2.5. February and April displayed similar TB PM2.5 levels, with Chiang Rai and Mae Hong Son provinces demonstrating the highest concentrations (approximately 30%) compared to other provinces. January witnessed comparatively lower TB PM2.5 levels; however, Tak province experienced a significant increase in TB PM2.5, exceeding other provinces and accounting for 31%. Finally, May exhibited the lowest TB PM2.5 concentrations across all provinces, although Chiang Rai province consistently maintained the highest level.

**Figure 5:**
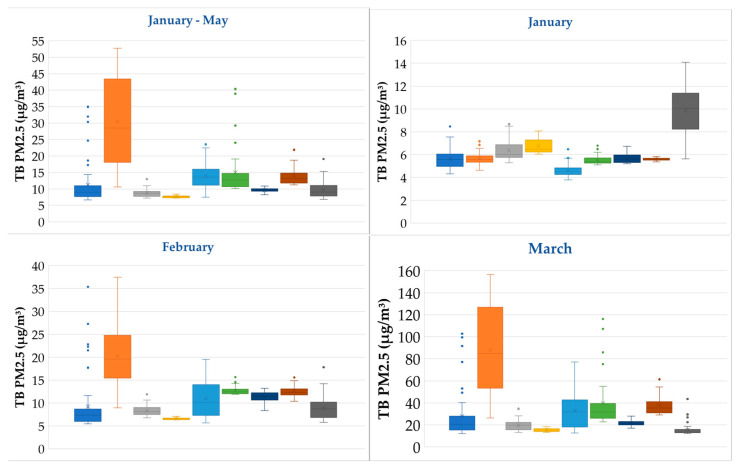

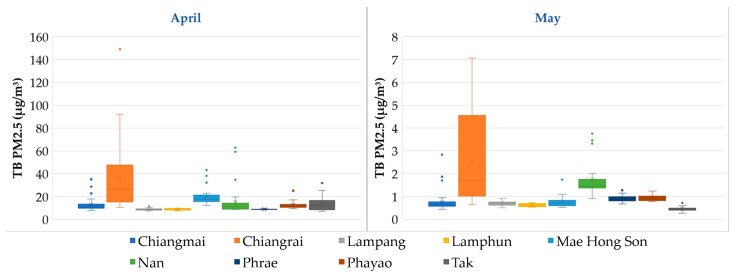
Boxplot of TB PM2.5 for the 9 provinces of northern Thailand.

The model output was analyzed as grid data, and the average concentration of TB PM2.5 for each province within the study area was depicted in box plots (Figure 5). These plots indicate that Chiang Rai exhibits the highest average TB PM2.5 concentrations. This province also demonstrates large spatial variation but lacks outlier values. In contrast, other provinces exhibit narrower ranges between their maximum and minimum TB PM2.5 concentrations, suggesting greater consistency within each province. However, it is notable that outliers are prevalent in several provinces, particularly Chiang Mai and Nan, where numerous extreme outliers occur. This implies that localized areas within these provinces experience significantly higher TB PM2.5 exposure than the provincial average. Lampang, Mae Hong Son, Phayao, and Tak display mild outliers, indicating some localized areas of elevated TB PM2.5 concentrations. Conversely, Lamphun and Phrae lack outliers, with minimal differences between their maximum and minimum values, suggesting a lower overall TB PM2.5 burden compared to the other provinces analyzed.

When considering the monthly average, January showed that TB PM2.5 crossed into the study area lightly. In most provinces, the amount of TB PM2.5 was not significantly different, and mild outlier values were detected in some areas. However, in Tak, the amount of TB PM2.5 was notably elevated, indicating a significant disparity compared to other provinces.

In February, an increase in TB PM2.5 was observed across all provinces. Notably, Chiang Rai exhibited the highest TB PM2.5 concentrations and a wide range of values. In Chiang Mai, several outliers were detected, approaching the maximum values observed in Chiang Rai. However, the majority of TB PM2.5 in Chiang Mai remained lower compared to other provinces. Mae Hong Son displayed the second-highest TB PM2.5 concentrations after Chiang Rai, with significant variations in value range. In contrast, most other provinces exhibited consistent TB PM2.5 concentrations, with a few outliers.

An analysis of TB PM2.5 concentrations during the crisis haze period in March revealed significant inter-provincial variations. Chiang Rai exhibited the highest levels, characterized by a notably wide range of values. Mae Hong Son followed with the second-highest TB PM2.5, also displaying a large value range. Both Chiang Mai and Nan experienced numerous severe outliers, some reaching Chiang Rai’s average value. Phayao recorded TB PM2.5 concentrations comparable to Mae Hong Son, exhibiting a consistent range with minimal outliers. Conversely, the remaining provinces displayed lower TB PM2.5 levels, with Tak being the only exception, exhibiting both severe and mild outliers.

In April, the TB PM2.5 levels and trends were consistent with those observed in March. Chiang Rai maintained the highest TB PM2.5 concentrations, characterized by a severe outlier and the most significant variation in values. Subsequently, Tak, Mae Hong Son, Nan, Chiang Mai, and Phayao followed in terms of TB PM2.5 concentrations. Notably, Chiang Mai, Mae Hong Son, and Nan experienced frequent occurrences of severe outliers, while Lampang, Lamphun, and Phrae consistently recorded substantially lower TB PM2.5 concentrations.

May exhibited the lowest impact from TB PM2.5 throughout the study period, coinciding with the end of the haze season. However, Chiang Rai consistently recorded the highest TB PM2.5 concentrations, demonstrating a significant deviation in values compared to other provinces. Nan followed with the second-highest TB PM2.5 concentrations. Notably, both Nan and Chiang Mai displayed a small number of severe outliers. Conversely, the remaining provinces experienced comparatively minimal TB PM2.5 concentrations within their respective provinces.

### 3.4. Impacts of Transboundary Biomass Burning PM2.5 on Local Air Quality and Public Health

**Figure 6:**
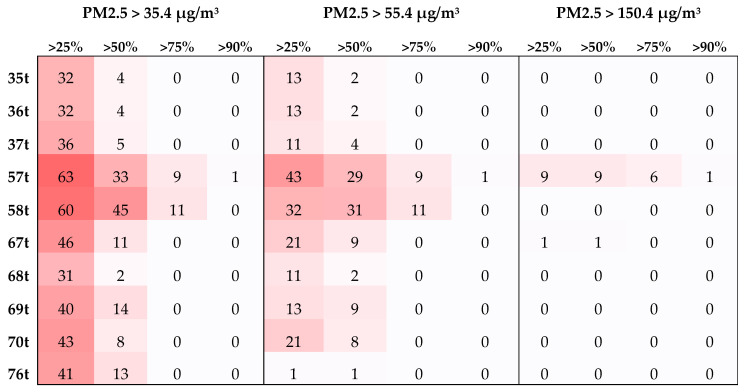
Number of days affected by transboundary (TB) PM2.5 at 10 PCD monitoring stations based on specified criteria. The darker red shaded indicates the greater number of days.

Based on the number of days characterized by the presence of transboundary biomass burning PM2.5 (TB PM2.5) across the 10 monitoring stations throughout the study period spanning 152 days, the report analyzes the occurrence of TB PM2.5 concentrations exceeding various thresholds across designated stations (Figure 6). The details are as follows.

Criteria 1: PM2.5 Concentration > 35.4 µg/m^3^ (Unhealthy for Sensitive Groups)

At 25% TB PM2.5, all stations consistently exceed the 35.4 µg/m^3^ threshold for a minimum of 31 days. Stations 57t (Chiang Rai) and 58t (Mae Hong Son) experience the longest durations, exceeding this threshold for over 60 days. Stations 67t (Nan), 69t (Phrae), 70t (Phayao), and 76t (Tak) follow with occurrences exceeding 40 days. Stations 35t–36t (Chiang Mai), 37t (Lampang), and 68t (Lamphun) show exceedances for around 30 days. For 50% TB PM2.5, this trend of consistent exceedance persists for all stations, with station 58t leading with 45 days and station 57t recording 33 days. Stations 67t, 69t, 70t, and 76t report approximately 10 days, while other stations exhibit fewer occurrences. As the threshold increases to 75% and 90%, the number of stations experiencing exceedances significantly decreases. Only stations 57t and 58t report occurrences, with durations of 9 and 11 days for 75%, respectively, and only station 57t recorded 1 day for 90%. When TB PM2.5 concentrations reach the Unhealthy for Sensitive Groups levels (>35.4 µg/m^3^), all stations experience a TB PM2.5 influence exceeding 25% for at least 31 days. Stations 57t and 58t exhibit consistently higher occurrences across all thresholds. Station 57t is the only station to experience TB PM2.5 exceeding 90%.

Criteria 2: TB PM2.5 Concentration > 55.4 µg/m^3^ (Unhealthy)

For PM2.5 proportions exceeding 25%, this pattern is observed at all stations. Notably, stations 57t and 58t emerged as focal points, recording 43 and 32 days, respectively. A similar trend was found at stations 67t and 70t, reaching 21 days. The remaining stations recorded approximately 10 days meeting this criterion, with station 76t registering a single instance. When considering the criterion of exceeding 50% TB PM2.5, this pattern was predominantly noted at all stations for less than 10 days. However, stations 57t and 58t again demonstrated elevated occurrences, enduring 29 and 31 days, respectively. As the threshold increased to 75%, occurrences were confined to stations 57t and 58t, with 9 and 11 days, respectively. At the 90% threshold, only station 57t exhibited a single day of this influence. This analysis underscores that all stations experienced continuous TB PM2.5 influence when TB PM2.5 concentrations reached Unhealthy levels (exceeding 50% for at least 1 day for all stations). Stations 57t and 58t stood out with significantly higher percentages, with station 57t being the sole station exceeding the 90% threshold.

Criteria 3: PM2.5 Concentration > 150.4 µg/m^3^ (Very Unhealthy)

Our analysis revealed that stations 57t and 67t displayed notable exceedances of this TB PM2.5 concentration threshold. Specifically, station 57t experienced 9 days where TB PM2.5 exceeded 25% of the total PM2.5 concentration, while station 67t recorded a single day. When considering the stricter threshold of 75% TB PM2.5, only station 57t exhibited occurrences, with a total of 6 days. Similarly, at the 90% threshold, only station 57t recorded a single day with such elevated influence. These findings highlight the consistently significant impact of TB PM2.5 at station 57t, especially during periods of Very Unhealthy levels. While station 67t also demonstrated a relatively high percentage of exceedances, its impact was notably less pronounced compared to station 57t. This analysis highlights the significant influence of transboundary biomass burning PM2.5 on local air quality and public health, particularly in specific monitoring stations that experienced high levels of exceedances.

## 4. Discussion

### WRF-Chem Model Evaluation

Our analysis of transboundary biomass burning PM2.5 (TB PM2.5) across 10 monitoring stations over a 152-day study period revealed significant spatial and temporal variations in PM2.5 concentrations. Chiang Rai province consistently exhibited the highest average TB PM2.5 concentrations, with substantial spatial variation but few outliers. Mae Hong Son also demonstrated high TB PM2.5 levels with significant variation. Conversely, Lamphun and Phrae recorded the lowest TB PM2.5 concentrations, with minimal differences between maximum and minimum values and no outliers. Notably, stations 57t (Chiang Rai) and 58t (Mae Hong Son) frequently exceeded health-relevant thresholds, particularly at the higher concentration criteria.

The findings indicate that transboundary biomass burning significantly impacts air quality in Northern Thailand, with certain provinces experiencing notably higher PM2.5 levels. This has important implications for regional air quality management and public health policies, highlighting the need for targeted interventions in the most affected areas. The consistent exceedances of health-relevant PM2.5 thresholds underscore the urgency of addressing transboundary pollution sources and implementing effective mitigation strategies.

Our results align with previous research, indicating the significant impact of transboundary biomass burning on air quality in Southeast Asia [32,33,34]. Similar studies have documented high levels of PM2.5 during biomass burning events, with substantial contributions from transboundary sources [35]. A study by Betha et al. (2013) [36] reported that biomass burning in Southeast Asia contributed significantly to PM2.5 concentrations in downwind regions, consistent with the spatial patterns observed in our study. More recent studies continue to support these findings. Additional studies also highlight the severe health impacts of PM2.5 from biomass burning. For instance, Karanasiou et al. (2021), Basith et al. (2022), and Krittanawong et al. (2023) [37,38,39] reported the toxicological effects of PM2.5 in affected populations, showing a clear link between biomass burning events and increased respiratory and cardiovascular diseases. Reducing biomass burning is essential to decrease PM2.5 exposure in many regions, including Europe [40], the United States [41], China [42], Southeast Asia [43], and northern Thailand [44]. This reduction is critical for mitigating health impacts associated with PM2.5 pollution.

One limitation of our study is the reliance on data from only 10 monitoring stations, which may not fully capture the spatial heterogeneity of TB PM2.5 across the entire region. Additionally, the study period of 152 days may not encompass the full variability of biomass burning events throughout the year. The use of model simulations, while robust, also introduces uncertainties related to model parameters and input data.

Despite these limitations, our study has several strengths. The comprehensive analysis of TB PM2.5 concentrations across multiple thresholds provides a detailed understanding of the pollution levels and their health implications. The use of grid data and box plots allows for a nuanced assessment of spatial and temporal variations, enhancing the robustness of our findings. Furthermore, the focus on transboundary pollution highlights an important environmental issue that requires coordinated regional efforts to address.

The public health implications of our findings are significant. Prolonged exposure to high levels of PM2.5, especially from biomass burning, is associated with adverse health outcomes, including respiratory and cardiovascular diseases. The high concentrations of TB PM2.5 observed in Chiang Rai and Mae Hong Son suggest a heightened risk for residents in these areas. Policymakers need to prioritize air quality management and public health interventions in these regions to mitigate the impact of transboundary biomass burning on local communities.

## 5. Conclusions

Our study reveals significant spatial and temporal variations in transboundary biomass burning PM2.5 (TB PM2.5) concentrations across northern Thailand, with Chiang Rai and Mae Hong Son provinces experiencing the highest levels. Stations 57t (Chiang Rai) and 58t (Mae Hong Son) frequently exceeded health-relevant PM2.5 thresholds, indicating a substantial public health risk. The findings align with previous research on the impact of transboundary biomass burning in Southeast Asia, emphasizing the need for targeted air quality management and regional collaboration to mitigate these effects and protect public health.

## Figures and Tables

**Table 1:** The details and coordinates for 10 air quality monitoring stations of PCD.

Station ID	Station Name	Latitude	Longitude
35t	Chang Pueak	18.8406330	98.9696610
36t	Sri Phum	18.7909205	98.9881062
37t	Lampang	18.2782510	99.5064470
57t	Chiang Rai	19.9092690	99.8233500
58t	Mae Hong Son	19.3047180	97.9709930
67t	Nan	18.7888780	100.7763590
68t	Lamphun	18.5671790	99.0385600
69t	Phrae	18.1289280	100.1623450
70t	Phayao	19.1638620	99.9027150
76t	Tak	16.7501080	98.5913130

**Table 2:** Configuration of the WRF-Chem model. (Applied from Chotamonsak and Lapyai, 2018 [20]).

Configuration	Details
Simulation period	January to May 2019
Domains	1 domain
Horizontal resolution	20 km (D01)
Vertical resolution	38 layers from 1000 to 10 mb
Metrological IC and BC	The National Centers for Environmental Prediction Final Analysis (NCEP-FNL)
Shortwave radiation scheme	Goddard shortwave radiation scheme
Longwave radiation scheme	The rapid radiative transfer model (RRTM) [21]
Land surface	The NOAH Land Surface Model [22]
Surface layer	Monin–Obukhov [23]
PBL	The Yon Sei University Scheme (YSU) [24]
Cumulus	The Grell–Devenyi ensemble [25]
Microphysics	Purdue Lin [26]
Aerosol model	The RADM2 SOGRAM aerosols model (direct and indirect effect)

**Table 3:** Average TB PM2.5 levels (µg/m^3^) for nine provinces in northern Thailand.

Province	January	February	March	April	May	AVR 5 Month
Tak	10.01 (31%)	9.00 (28%)	15.83 (42%)	13.61 (39%)	0.45 (2%)	9.77 (31%)
Chiang Mai	5.66 (20%)	9.20 (25%)	27.72 (45%)	12.55 (29%)	0.74 (3%)	11.20 (29%)
Mae Hong Son	4.65 (17%)	10.92 (32%)	32.87 (55%)	19.76 (44%)	0.75 (3%)	13.80 (37%)
Chiang Rai	5.69 (18%)	20.38 (41%)	86.53 (68%)	31.25 (37%)	2.38 (7%)	29.41 (45%)
Phayao	5.59 (17%)	12.56 (27%)	38.01 (47%)	12.92 (21%)	0.92 (3%)	14.03 (28%)
Lampang	6.37 (21%)	8.38 (21%)	19.62 (37%)	8.72 (23%)	0.70 (3%)	8.76 (23%)
Phrae	5.65 (19%)	11.26 (25%)	21.76 (36%)	8.84 (24%)	0.90 (3%)	9.65 (24%)
Nan	5.49 (17%)	12.73 (27%)	38.49 (44%)	14.83 (28%)	1.71 (6%)	14.68 (29%)
Lamphun	6.74 (23%)	6.60 (17%)	15.36 (35%)	8.98 (25%)	0.62 (3%)	7.67 (22%)
AVR 9 Provinces	6.21 (20%)	11.22 (27%)	32.91 (46%)	14.61 (30%)	1.02 (4%)	13.22 (30%)

The percentage (%) represent the proportion of the average TB PM2.5 entering each province, calculated by comparing between Case 1 and Case 3 for each provincial boundary.

## Data Availability

All data generated or analyzed during this study are included in this published article.
